# Abuse of Licit and Illicit Psychoactive Substances in the Workplace: Medical, Toxicological, and Forensic Aspects

**DOI:** 10.3390/jcm9030770

**Published:** 2020-03-12

**Authors:** Ricardo Jorge Dinis-Oliveira, Teresa Magalhães

**Affiliations:** 1 IINFACTS—Institute of Research and Advanced Training in Health Sciences and Technologies, Department of Sciences, University Institute of Health Sciences (IUCS), CESPU, CRL, 4585-116 Gandra, Portugal; 2 Department of Public Health and Forensic Sciences, and Medical Education, Faculty of Medicine, University of Porto, 4200-319 Porto, Portugal; 3 UCIBIO-REQUIMTE, Laboratory of Toxicology, Department of Biological Sciences, Faculty of Pharmacy, University of Porto, 4050-313 Porto, Portugal; 4 Fidelidade—Companhia de Seguros, SA, R. Direita de Campinas 324, 4100-207 Porto, Portugal

**Keywords:** occupational medicine, forensic medicine, insurance medicine, psychoactive substances, safety, clinical, forensic, law, ethics

## Abstract

About one-third of adult life is spent in the workplace. The use of psychoactive substances is a major preventable cause of morbidity and mortality. The consumption of psychoactive substances during or outside working hours greatly increases the frequency and severity of labor accidents, as well as the workers’ poor general state of health and productivity, implying higher costs for enterprises. It is the responsibility of organizations to ensure the safety and health of their workers. These cannot be limited to traditional routine clinical exams, as other aspects also have an impact on health. Thus, prevention and intervention in the consumption of psychoactive substances (e.g., ethanol, opioids, central nervous system stimulants or depressants, hallucinogens, *Cannabis* derivatives, dissociative substances, and inhalants) in labor activity should be considered as an investment of organizations and not as a cost, in view of the professional, personal, and family advantages for workers and employers, with a potential impact on productivity, security, health, and quality of life at work. Despite the extensive literature on the subject, each article generally focuses on one or another aspect of a very specific nature, not tackling the problem in a holistic way by confronting clinical, safety, and legal issues. This article presents a reflection on the legal, laboratorial, clinical, ethical, forensic, and safety concerns related to the consumption of psychoactive substances in the workplace, and can be a cross-cutting contribution to occupational medicine, forensic medicine, and insurance medicine, as well as for entrepreneurs, lawyers, judges, workers, and technicians from the public and private sectors that develop projects in this area. This discussion is based on general principles established internationally and highlights the role of the occupational healthcare system and other decision-making actors in the prevention and supervision of workplace psychoactive consumption.

## 1. Introduction

Workplaces reflect, to a certain extent, the widespread presence of ethanol and other psychoactive substances in society and the type of work, but sometimes both. There has been global growth in the use of workplace drug testing as a response to drug-related risks to safety and productivity [1,2]. Depending on the countries, the labor sectors, and the professions, statistics suggest high percentages of workers suffer from or are at risk of becoming dependent on ethanol [3]. Civil construction workers, transport workers, hotel staff, barmen, catering workers, farmers, and workers in the primary sectors are particularly affected by this problem, especially in terms of ethanol [3]. Additionally, ethanol-related indicators are often inversely proportional to the level of literacy, meaning that the higher this level is, the less likely workers are to become drunk [4]. However, in some countries, excessive ethanol or other substance consumption is clearly observed among some higher professional groups such as doctors and managers [5,6]. Regarding the consumption of other psychoactive substances in the workplace, the information is scarcer, with *Cannabis* being more prevalent among younger workers, whereas cocaine is more prevalent among highly skilled workers such as managers [7]. Indeed, because workplace drug testing is often performed on-site by occupational physicians, a global statistic spectrum is hard to obtain [8].

The consumption of psychoactive substances at work depends on the combination of multiple factors, some of them linked to individual characteristics and lifestyles, and others being of a professional nature, related for example to the typology of work, rhythms and cadences, shift work, and stress, among others [9,10].

It is obvious that the prevention and deterrence of problems associated with the use of psychoactive substances should be a global intervention involving the participation of all decision-making actors in the organization bodies, namely, occupational medicine, occupational safety and health, human resources, social action services, intermediate and direct leadership, workers’ representatives, and workers themselves. Detection may or may not be part of the organization’s health and safety policy procedures. In order to create a program that contemplates psychoactive substance testing, the underlying policy, objectives, and rights and responsibilities of all parties involved should be made explicit. Moreover, the analysis of results and the relationship with the worker must respect confidentiality, cooperation, mutual commitment, and capacity building. The final goals are beneficial both for employers and employees and include the increase of workplace productivity by reducing absenteeism, presenteeism, and workplace accidents, as well as raising awareness of the toxic effects of psychoactive substances and their consequences in workers’ performance by adopting a more healthily behavior during and outside working hours [9,11,12].

This article presents a reflection on the legal, laboratorial, clinical, ethical, forensic, and safety concerns related to the consumption of psychoactive substances in the workplace, and can be a cross-cutting contribution to occupational medicine for businessmen, lawyers, juries, workers, and laboratory technicians that act in this area. We also aimed to discuss the best practices to be followed by laboratories providing workplace drug testing services. 

## 2. Methods

A narrative review was performed by searching articles in English, French, Spanish, and Portuguese in PubMed, Scopus, Web of Science, and PsycINFO concerning legal, laboratorial, clinical, ethical, forensic, and safety concerns of workplace drug testing, without time limit. Besides these inclusion criteria, additional reports were obtained from the references of the articles identified in the original search. International reports from the World Health Organization; European Union; International Commission on Occupational Health; and legislation on psychoactive substance workplace consumption, testing, and prevention were also reviewed. Specifically, Portuguese legislation, under the European Union regulation, was used as a starting point and, regardless the jurisdiction, broad and transversal aspects general and useful for occupational medicine were discussed. The effects in workplace performance of ethanol, opioids, central nervous system stimulants or depressants, hallucinogens, *Cannabis* derivatives, dissociative substances, inhalants, and examples of new psychoactive substances were also reviewed. From this search, 192 documents were obtained and 105 were ultimately considered for the final version of the manuscript. The remaining documents were excluded if they focused on specific points of certain enterprises or on a very specific point of a country not considered useful for a broad discussion, or when those aspects do not fit well in the scope of occupational medicine, such as technical and analytical details of toxicological methods.

## 3. Psychoactive Substances and Occupational Risks

Psychoactive substances are those that act mainly on the central nervous system, where they alter brain function, resulting in temporary changes in perception, mood, consciousness, and behavior. The effect depends not only on the specific substance but also on the dose, previous consumption, tolerance, comorbidities, mixture with other substances, and route of administration, among other reasons. In a general overview, all psychoactive substances have, to a higher or lesser extent, a dysfunctional effect on work capability. Considering the clinical usefulness and their most important effects at the level of the central nervous systems at usual doses, psychoactive substances are classified into several groups, which are discussed below regarding their repercussions in terms of workplace performance.

**Opioids** are mainly used as analgesics and antitussives [13,14,15,16,17]. Physical work and high psychosocial work demands, excessive repetition of tasks, awkward postures, and heavy lifting are all known workplace risk factors for musculoskeletal pain and consequently lead to administration of opioids, according to prospective studies [18,19]. Interestingly, a recent randomized trial demonstrated that treatment with opioids was not better to treatment with nonopioid medications for improving pain-related function over 12 months in moderate to severe chronic back pain or hip or knee osteoarthritis pain [20]. Part of this group are natural compounds extracted from opium such as morphine, codeine, and thebaine; semisynthetic compounds such as heroin, oxycodone, hydrocodone, oxymorphone, etorphine, and hydromorphone; and the synthetics tramadol, tapentadol, meperidine (or pethidine), methadone, fentanyl, and pentazocine. Work-related injuries have been identified as a factor in the rise of opioid dependence and opioid-related overdoses [21,22]. Opioid use has also been associated with the risk of motor vehicle accidents in commercial drivers [23]. Indeed, several adverse effects are possible risk factors for workplace performance, namely, those mediated by µ-receptors, such as sedation, respiratory depression, suppression of cough reflex, sweating, euphoria, dysphoria, confusion, insomnia, agitation, fear, hallucinations, drowsiness, motor decoding and mood swings, miosis, and dependence [17,24,25].

Cocaine, amphetamines (i.e., *d*-amphetamine, *d*-methamphetamine, methylphenidate, 3,4-methylenedioxymethamphetamine), caffeine, nicotine, and ephedrine are **stimulants of the central nervous system** [26,27,28]. Xenobiotics belonging to this group exhibit, in general, a pronounced stimulating effect in the central nervous system. They reduce the feeling of mental (i.e., increased alertness) and physical fatigue (i.e., increased motor activity) and cause dependence [29,30]. Of these, the most consumed psychoactive substance is caffeine, which is present in coffee, tea, and chocolate, as well as in numerous foods, drugs, and beverages, as it increases energy and concentration, with no major negative labor effects having been reported [31], and it moreover has been linked to a reduced suicide risk [32]. In addition, considerable research suggests that nicotine enhances cognitive control-related processes (e.g., attention, memory) among nicotine-deprived smokers, both in terms of behavior and neural indices [33]. Indeed, smokers deprived of nicotine (e.g., 12 h smoking abstinence) exhibit reduced cognitive-attentional functioning [34]. Employees who tested positive for cocaine were four times more likely to be categorized as absentee employees and two times more likely to be terminated from employment than those who tested negative [35]. Comparing the consumers of amphetamines with those of other illicit psychoactive substances, it was found that they had higher absenteeism due to disease and/or accidents, as well as a higher incidence of behavioral risks. If consumption occurs outside working hours, the period of depression and asthenia and cognitive alterations may arise when they resume work activity. Low-to-moderate doses of stimulants (e.g., amphetamine, caffeine, modafinil) have been reported to be effective countermeasures for mood and performance decrements caused by sleep deprivation and fatigue [36,37].

More than 61 **cannabinoids** have been identified among more than 400 compounds documented in *Cannabis sativa* [16,38,39,40]. The most important forms are delta-9-tetrahydrocannabinol (Δ^9^-THC; main psychoactive constituent), Δ^8^-THC (almost as active as the previous one but at lower concentrations), cannabinol (low activity, but at high concentrations), and cannabidiol (not psychoactive, but in high concentrations). Δ^9^-THC is the most widely abused illicit psychoactive substance and the potency of the derivative (e.g., marijuana, hashish, hashish oil) depends on the percentage of this xenobiotic [16,39,40,41]. Δ^9^-THC produces euphoria followed by relaxation, distortion in space and time, hallucinations (in higher doses than those normally found clinically), changes in short-term memory, motor incoordination, behavioral disinhibition, concentration and learning issues, decreased appetite, and in high doses paranoid psychosis. It possesses a low risk of psychological dependence and its abstinence is typically characterized by insomnia [42]. Marijuana cigarettes containing low-to-moderate Δ^9^-THC concentrations can decrease some night shift-related performance and mood disruptions [43,44]. Occupational medicine additional concerns come from the use of medicinal *Cannabis* [45,46,47]. Workers who have been authorized to use *Cannabis* should be required to report any change in product, dose, frequency, and timing of use or route of administration, and an occupational physician trained and knowledgeable on the impact and evaluation of potentially impairing substances in the workplace should be included [47]. Moreover, new psychoactive substances, such as synthetic cannabinoids, are now an additional concern to deal with [48]. Indeed, these compounds also bind to cannabinoid receptors and abuse can cause anxiety; confusion; hypertension; psychosis; hallucinations; tachycardia; seizures; and, in severe cases, it can lead to death [49,50,51].

**Hallucinogens**, such as the diethylamide of lysergic acid (LSD), mescaline, psilocybin, and psilocin, also have a pronounced effect on workplace performance [52,53]. Hallucinogens intensify sensory experiences and lead to behavioral alterations, delusions, as well as emotional lability at the time of and after consumption, confusional states, and *flashbacks* (i.e., reviving experiences). Chronic consumption can lead to depression, violent behavior, anxiety, and alteration in the perception of time, but the risk of dependence is absent.

The **central nervous system depressant** group mainly includes psychotropic drugs, notably the illicit use of anxiolytics, sedatives, and hypnotics, such as benzodiazepines; barbiturates; and non-benzodiazepine hypnotics such as zolpidem and ethanol [54,55]. Of all substances, ethanol is the one with the highest negative impact on work [9,56]. Globally, ethanol is the world’s number one risk factor for ill-health and premature death amongst the 25- to 59-year-old age group, the core of the working-age population [57]. Moreover, in the USA, ethanol-induced impairment directly affects an estimated 15% of the workforce and causes more than 22% of the deaths as a result of injuries at work [58]. The main labor consequences of ethanol consumption are slow reaction time, motor incoordination, decreased visual acuity, emotional lability, reduced concentration, lower intellectual ability, behavioral changes, attendance/punctuality problems, lower productivity, absenteeism, presenteeism, workplace injuries, and higher employee turnover [59]. Several studies have shown that workers who drink the most are more often absent from work due to drinking [60], and they more often report alcohol-related presenteeism and inefficiency at work due to alcohol use [61]. Ethanol is anxiolytic/disinhibiting and has a high potential for abuse. Withdrawal leads to sweating, nausea, tremor, insomnia, decreased appetite, restlessness, aggression, anxiety, and eventually hallucinations. It is often used in conjunction with other substances to enhance the overall effect. Major international organizations such as the World Health Organization, the Council of the European Union, and the International Labor Office advertise the need as priority to actualize policies and programs focused on the issue of ethanol and work.

Among psychoactive drugs, benzodiazepines are the most prescribed drugs, especially as anxiolytic, sedative, or hypnotic drugs, and less often as antiepileptics and/or muscle relaxants [55]. Benzodiazepines have largely replaced barbiturates as they are safer drugs with fewer enzyme-inducing effects, and thus less severe interactions, less severe withdrawal symptoms, and a broad therapeutic margin [62]. However, persistent and longer than recommended use and self-medication is a reality. Benzodiazepines are distinguished between anxiolytic and hypnotic [24,55]. This distinction is somewhat artificial because all are anxiolytic, and all can change sleep as long as certain doses are reached [55,63]. What distinguishes them, however, is that so-called hypnotic benzodiazepines are potent drugs that can, therefore, modify sleep conditions at relatively low doses, whereas so-called anxiolytic benzodiazepines are less potent, allowing for a “therapeutic window” to exist where anxiolytic action can be obtained without significantly interfering with sleep [55]. All benzodiazepines may induce tolerance, dependence, and addiction, but to a lesser extent than barbiturates [62]. Short-acting benzodiazepines have the highest potential to induce dependence, and withdrawal syndrome may even occur when discontinuation is abrupt. The abuse of these drugs is generally higher in females and older workers. The toxic effects of benzodiazepines on quality of work are mostly related to the central nervous system depressant effects, particularly sleepiness, motor incoordination, and impaired thinking [64]. They have a significant impact on motor vehicle driving, workability, and interpersonal relationships, and thus their use should be taken into account in these circumstances [65]. They can also cause short-term memory impairment, that is, diminished ability to learn new information, leaving already learned information intact.

Among **dissociative drugs**, phencyclidine and ketamine were originally developed as dissociative general anesthetics, capable of promoting sensory loss and analgesia, amnesia, and paralysis, generating an intense sensation of dissociation of the environment but without real loss of consciousness and protective reflexes [66,67]. Only ketamine is still available in therapy due to the high frequency of delusions and hallucinations observed postoperatively with phencyclidine [67,68,69,70]. The most classic adverse effects of ketamine are delirium, hallucinations, tachycardia, mild respiratory depression, confusion, irrationality, violent or aggressive behavior, dizziness, ataxia, slurred speech, delayed reaction time, euphoria, altered body image, analgesia, amnesia, and coma [66,67].

## 4. Psychoactive Substances and Occupational Consequences

Employers, for their part, have a broad range of responsibilities, and it is the employer’s obligation to ensure the individual and collective health of workers for the proper functioning of all work activities. Psychoactive substance abuse is a major preventable cause of morbidity and mortality and has direct impacts on workability [71]. Staying in the workplace under the influence of psychoactive substances depends on the combination of multiple factors, some linked to individual characteristics and lifestyles, and others of professional nature, such as work typology, rhythms and cadences, irregular working hours as occurs in shift work, stress, and psychological harassment, among others. The following are some of the consequences of the occupational psychoactive substance consumption and thus justify why employers aim for a ‘‘drug-free’’ workplace [72,73,74]: (i) increase in the rate of presenteeism (i.e., being present at workplace in an impaired state) and absenteeism that affects professional performance promoting errors, and hence the competitiveness and productivity of the enterprise, as well as the country’s own wealth; (ii) creation of a negative image, leading to discrediting and despising of the organization; (iii) negative effect on the equipment integrity and therefore a potential cause of financial losses; (iv) negative effect on the workers’ physical, psychosocial, and behavioral integrity; (v) risk of neglect and reduced decision-making and motor coordination, consequently leading to a higher number of errors and accidents and therefore costs (e.g., insurance premiums); (vi) workers being more often involved in conflict, violent behavior, and theft, and being more frequently the subject of complaint by coworkers who may also see their physical integrity or even their own lives affected as a result of lack of care or discernment, decreased alertness, or altered perceptions or judgements from others being under the influence of psychoactive substances; and (vii) workers tending to be non-punctual (arriving at work later and leaving early) than the rest of the working population, putting a greater strain on coworkers by introducing additional tasks that still need to be done.

## 5. Legal Aspects

Aiming at a legal interpretation of this theme, it is important to make clear what is an occupational accident and workplace. Although, these concepts may vary with the different legislation of each country or state, regardless the jurisdiction, these are broad concepts in most legislations. We used Portuguese legislation as a starting point for this approach.

The Portuguese Law no. 98/2009 of 4 September regulates the regime of compensation for occupational accidents and diseases, including occupational rehabilitation and reintegration, pursuant to article 284 of the Labor Code, approved by Law no. 7/2009 of 12 February. In accordance with Article 8, an ***occupational accident*** is one that occurs at the place and time of work and that directly or indirectly results in bodily injury, functional disturbance, or illness resulting in reduced working or earning capacity, or death. Therefore, it is an event that has a professional factor for its occurrence, including acts of violence that occurred within the scope of the workplace concept. Its major differences from the concept of occupational disease, in which occupational factors are also determinant, include (i) very short time (at most a few minutes), usually sudden and unexpected (acute); (ii) easy identification of the cause (professional); and (iii) easy identification of the lesion.

The concept of the ***workplace*** means any place where a worker is or should be by virtue of his/her work and that are directly or indirectly under the employer’s control, including road accidents during work-related activities. The term “working time beyond the normal working period” is defined in Portuguese law as the time that (i) precedes its commencement, including preparatory acts; (ii) follows, including related acts; (iii) resulted from normal or forced interruptions of work. Article 9 extends the concept, and also considers a workplace accident as one that occurs (i) on the way to or from the workplace in the routes normally used and considering the period of time usually spent by the worker in those routes: between any of their workplaces if they have more than one job (the destination employer being the responsible for the accident), between his/her habitual or occasional residence and the workplace or the place of payment or the place where they will receive any kind of assistance or treatment by reason of a previous accident, or between the workplace and the place of meal and between the new place defined by the employer for a specific work and the usual workplace or his/her habitual or occasional residence. It is also considered a work accident when the normal route has been interrupted or changed in order to satisfy workers’ needs, as well as due to force majeure or due to a fortuitous event (ii) while performing spontaneous services that may result in economic gain for the employer; (iii) at and outside the workplace while representing workers; (iv) at the workplace, when attending a training course, or outside the workplace, if the express permission from the employer for such attendance was obtained; (v) at the place of payment of remuneration and while staying there for that purpose; (vi) at the place where the worker should receive any form of assistance or treatment due to a previous accident and while staying there for that purpose; (vii) while job searching during the hours granted by law to workers with process of termination of the current work contract; and (viii) off-site or working time when verified in the performance of services determined by the employer or consented by him/her.

### 5.1. Workplace Drug Testing

The legislation applied to workplace drug testing also has some differences among countries [75]. Indeed, a multinational company may not be able to implement the same procedure in all its offices. In this work, we focused upon and discussed general consentaneous aspects that need to be followed in the application of a program for workplace drug testing; the Portuguese legislation was used as a platform for critical reflection. This approach is even more interesting because by adopting a radical step, in July 2001 Portugal became the first country in the world to decriminalize the possession for consumption of all illicit substances (Law no. 30/2000 of 20 November). Rather than being arrested, those caught with a personal supply are obliged to undergo rehabilitation treatment.

Although there are no specific propositions referring to psychoactive substance testing in the workplace, the Portuguese law appoints a set of very strict general rules regarding the worker’s health. Indeed, the human life (Article 24); the moral and physical integrity of persons (Article 25); the right to preserve the intimacy of private life (Article 26); the right to the protection of personal data and the use of computers (Article 35); and the rights of all workers (regardless of age, sex, race, citizenship, territory of origin, religion, or political or ideological convictions) to have a job and work under conditions of hygiene, safety, and health, as well as the rights to have assistance and fair rehabilitation when they are victims of workplace accidents or occupational disease (Article 59) are rights constitutionally enshrined in the Constitution of the Portuguese Republic. Moreover, in Europe, any regulation must conform to the European Convention for the Protection of Human Rights and Fundamental Freedoms. The latter warranties for a person’s right to privacy, which states that everyone has the right to his/her private and family life, his/her home, and his/her correspondence, and that public authorities must not interfere with the exercise of this right, except if the interests of national security, public safety, or the economic well-being of the country are at risk. On an international level, the matter might be covered by Universal Declaration of Human Rights (Article 12), which states that no one shall be subjected to arbitrary interference with his/her privacy.

The use of psychoactive substances in the workplace (or during working hours) is usually governed by health and safety laws that address the potential health and safety risks for themselves or co-workers [75,76,77]. Therefore, these laws usually legitimate the fact that the tests should be restricted to categories of workers whose activity may endanger their physical or third party integrity and make employers responsible for preventing psychoactive substance use in the workplace and impose them to carry out risk assessments and preventive measures [78]. The real question is how we can fulfil the purposes of the law, namely, the following Portuguese legislation: (i) Articles 15 (employer’s general obligations), 16 (simultaneous or successive activities in the same workplace), and 17 (worker’s obligations) of the legal regime promoting occupational safety and health (Law no. 102/2009 of 10 of September); (ii) Articles 19 (medical tests and examinations), 99 (internal company rules), 281 (general principles on safety and health at workplace), 282 (information, consultation, and training of workers), 283 (accidents at work and occupational diseases), and 284 (regulation of prevention and reparation) of the Labor Code (Law no. 7/2009 of 12 February); (iii) Decree-Law no. 4/2015 of 7 of January (Code of Administrative Procedure); and (iv) the General Data Protection Regulation 2016/679 of the European Parliament and of the Council of 27 of April of 2016. Besides these legislations, specific regulations of each enterprise should be also taking into account. The Portuguese State, through the Labor General Inspection, the Directorate-General of Health, and the National Protection Centre against Professional Risks, proceeds to inspections to see if the rules are followed.

#### 5.1.1. The Legal Regime for the Promotion of Health and Safety at Work (Law no. 102/2009 of 10 September)

The legal regime for the promotion of health and safety at work (Law no. 102/2009 of 10 September) states that occupational safety and health must be based on a correct and permanent risk assessment and be developed according to principles, policies, standards, and programs, focusing on the promotion and monitoring of occupational health and the enhancement of technical and scientific research in the field of safety and health at the workplace, particularly the emergence of new risk factors (Article 5). In Portugal, the existence of in-house occupational safety and health services is mandatory in organizations with more than 400 workers or in those with more than 30 workers if they are exposed to high-risk activities. In cases where the enterprise does not have an internal occupational health and safety service, this must be assumed by the external entities that provide occupational health and safety services (Article 78).

There are several general obligations that the employer must continuously and permanently fulfil (Articles 15 and 18): (i) ensuring safety and health in the workplace, (ii) identifying all foreseeable risks in all activities of the organization, (iii) mitigating monotonous and repetitive work, and (iv) reducing psychosocial risks. These concerns should be balanced accordingly to the risks to which the workers are potentially exposed to and are recognized as causes for psychoactive substance use, which in turn is a risk factor for accidents and enhancer of work-related diseases. Nevertheless, it should be borne in mind that employers cannot legitimately invoke the obligation to carry out psychoactive substance screening tests to accomplish their duties of ensuring the health of workers (Article 108). Indeed, the obligation to perform clinical exams (i.e., for admission, periodic, or occasional) is duly specified in the legislation and its purpose is aimed at attesting and evaluating the physical and mental fitness of the worker to perform the activity, as well as possible repercussions. In other words, compulsory psychoactive substance testing undermines the rights, freedoms and personal guarantees enshrined in the Constitution of the Portuguese Republic, namely, the right to personal integrity (Article 25) and the right to privacy reserve (Article 26). This means that there will be no justification for drug testing in all (or random) workers in an organization, but only for those whose job requires high skills or involves considerable risk to themselves or other workers and in all who show manifested and serious signs of being influenced by psychoactive substances [79]. Moreover, it should be mentioned that the existence of an internal regulation that considers a program for workplace drug testing cannot be in itself a just cause for dismissal because it is not provided by law and violates the principle of job security and the fundamental right of workers accordingly the Article 53 of the Constitution of the Portuguese Republic.

On the other hand, it is also the worker’s obligation to comply with the occupational safety and health requirements provided by legislation and collective labor regulation instruments, as well as for instructions determined for that purpose by the employer (Article 17).

#### 5.1.2. The Labor Code (Law no. 7/2009 of 12 February)

Regarding the Portuguese Labor Code (Law no. 7/2009 of 12 February), the article 281 et seq. focuses on the prevention and reparation of accidents and occupational diseases in the workplace; workers have the right to provide work in safe and healthy conditions and should respect the occupational safety and health requirements laid down by law or collective labor regulation instruments, or those determined by the employer, and the employer should apply the necessary measures to provide such environments.

The carrying out of medical tests and examinations (Article 19) is within the scope of the organization of occupational safety and health services and must respect citizens’ rights, freedoms, and guarantees. Regarding the detection of psychoactive substance use, this may or may not be part of the organization’s health and safety policy. The most common procedure is to perform drug testing in those workers randomly nominated by the computer, as well as those appointed by the occupational physician, or to those who request according to the rules of procedure. As mentioned above, random drug testing has been prone to controversy because employers must ensure that every aspect of their policies is rooted in scientific evidence, linked rationally to the goal of workplace safety, and are ethically justifiable [79]. Some organizations also advocate testing following an accident of specific consequences (e.g., fatalities, injuries that require anyone to be removed from the scene for medical care, damage to vehicles or property above a specified monetary amount) in order to determine whether the abuse of psychoactive substances were a factor. Of course, a positive test for psychoactive substances cannot prove that this was the cause of the accident. 

Considering the possibility of creating a program that includes toxicological analyses for detection of psychoactive substances, this must comply with the legal rules in force and be part of an internal regulation according to Article 99. This regulation should spell out the underlying policy, objectives, and rights and responsibilities of all parties involved, as well as issues regarding the protection of personal data, namely, assuring the right of the worker to privacy, the need for his/her consent to perform the tests, and the preventive and non-sanctioning character of the drug tests. These are important topics specially designed to increase the sensitivity of health and safety professionals to this problem and encourage them to raise awareness of them.

Screening tests for psychoactive substance use are restricted to the occupational physician or, under his/her guidance and control, to other health professionals obliged to professional secrecy (e.g., occupational nurses) and trained to use the kits for toxicological analysis. The same is true for the results of the tests because this represents health information. Therefore, clinical data can only be known to health professionals from the occupational medicine team, who are subject to confidentiality, and any hypothetic witnesses nominated by the worker.

In order to comply with professional secrecy and to guarantee the confidentiality of information resulting from medical examinations, the occupational physician must record results with “generic terms”, namely, the worker (i) is fit (i.e., does not consume), (ii) is “fit with restrictions”, or (iii) is temporarily unable (i.e., consumes) to perform his/her duties. According to no. 2 of Article 17 (on the protection of personal data) of the Labor Code, at no time should the physician report the test results to the employer. Only in this way is the adequacy of the preventive and deterrent measures ensured and it represents a very serious offence the disrespect of these directives. Accordingly to Article 195 of the Portuguese Penal Code, the disclosure of other people’s secrets, known to someone on account of his/her state, job, employment, profession (e.g., physician), or arts, is punished by a term imprisonment of 1 year or fine until 240 days. Moreover, the physician also follows the Deontological Code of the Medical Association, which compels him/her to professional secrecy (Article 71).

It is also made clear by the Labor Code (Article 19), in addition to the situations provided in occupational safety and health legislation, that the employer cannot, for admission purposes or job maintenance, require the applicant or worker to perform or present tests or medical examinations of any nature (such as the results of psychoactive substance use tests), or prove physical or mental conditions and therefore health. An exception occurs when these examinations aim for the protection and safety of the worker or third parties, or when the demands inherent to the activity justify it. In these cases, the reasons must be provided in writing to the job seeker or worker. Therefore, conducting screening tests for psychoactive substance use will only be legitimate in exceptional cases where health, welfare, worker, employer, or third-party concerns are at stake. In cases of job admission, even if screening tests for psychoactive substance can be justifiably performed in candidates, the possibility of the job applicant stopping drug use several days before testing should be born in mind. However, the screening will no longer be legally acceptable on the principles of proportionality, appropriateness, and reasonableness when there are no objective grounds to perform them in order to assure safety to the workers, service users, or the wider community, or when, from this point of view, the risks are minimal.

It is also clear that a job applicant or worker who has provided personal information has the right to control his/her personal data, to be aware of its content and the intended purpose, and to ask for its correction and update. 

## 6. Diagnosis of the Influence of Psychoactive Substances and Toxicological Analysis

It is fundamental to have the substances that will be monitored clearly defined in the rules of procedure. Suspicion of psychoactive substance consumption can be made at various levels, of a subjective and objective nature. The loss of productivity and decreased quality of work, lack of punctuality and absenteeism, indiscipline and inappropriate behaviors, and the increase in workplace accidents are warning signs that cannot be neglected. However, these signs of suspicion should be part of a broader clinical and laboratory evaluation under the responsibility of occupational medicine and, in some cases, with the contribution of insurance medicine as well as forensic medicine.

Occupational medicine is a medical specialty concerned with the maintenance of health in the workplace, including prevention and treatment of diseases and injuries. In other words, the aim of occupational medicine focuses on workers’ health. Accordingly, the International Code of Ethics for Occupational Health Professionals published by the International Commission on Occupational Health (ICOH) states, “the aim of occupational health practice is to protect and promote workers’ health, to sustain and improve their working capacity and ability, to contribute to the establishment and maintenance of a safe and healthy working environment for all, as well as to promote the adaptation of work to the capabilities of workers, taking into account their state of health” [80].

Many enterprises establish a drug policy with little or no structure for drug testing, namely, (i) quality control, (ii) systematic confirmation of positives, and (iii) procedures to accomplish the chain of custody. Indeed, procedures that ensure the chain of custody compliance are of utmost importance in toxicological analyses. Moreover, drug testing is usually performed on-site by occupational physicians, who are usually not familiarized with analytical toxicological aspects. In Europe, the European Guidelines for Workplace Drug Testing have been prepared and updated by the European Workplace Drug Testing Society (EWDTS) for different samples. These guidelines are designed to [81] (i) establish best practice and standard procedures whilst allowing individual countries to operate within the requirements of national customs and legislation, (ii) ensure that the entire drug testing process is conducted to give accurate and reliable information about a donor’s drug use, (iii) maintain the legal defensibility and scrutiny either by an employment tribunal or a court of law, (iv) protect the dignity of the specimen donors and the validity of the specimen, (v) define common and critical quality control procedures for laboratories, and vi) help in the interpretation of the analytical results. Guidelines for collection of biological samples for toxicological analysis were recently published [82] and have largely conformed to the EWDTS guidelines, focusing on sample collection and testing in urine, hair, and oral fluid. Our work focused only on specific points relevant to the interpretation of workplace drug testing [75,81]. Trained personnel, who do not need to specifically be healthcare professionals, are required [81]. All samples must be kept for an agreed period or in respect of the national legislation of each country to allow rebuttal if any judicial sues are made regarding the obtained results. After the agreed time, the laboratory may discard the sample if the customer did not request the laboratory to retain the sample for an additional period. Samples must be retained within the secure laboratory area until the disposal date agreed with the customer [83].

### 6.1. Interpretation of Ethanol Results

The alcoholaemia is usually performed by quantitative breath analysis, using an alcohol meter (duly calibrated). In Portugal, there is a model certified by the Portuguese Institute of Quality that is based on the theoretical relationship defined in Law no. 18/2007 of 17 May, assuming that 1 mg/L in breath alcohol concentration (BrAC) is equal to 2.3 g/L of blood alcohol concentration (BAC) [65]. Counterproof should always be available to confirm results and should be provided by a referenced toxicology laboratory and by using a different technique and chemical principle from that of the screen test in order to ensure reliability and accuracy. Real BAC can be quantified by blood collection and further analysis by using gas chromatography with a flame ionization detector and a headspace system. The direct determination of ethanol itself in hair is not possible due to its volatility and its potential absorption from external sources [54]. Instead, the minor ethanol metabolites ethyl glucuronide (EtG) and/or fatty acid ethyl esters (FAEE) can be measured in hair by GC or LC coupled to MS/MS as a direct alcohol consumption marker [84].

There are no legally defined values for ethanol blood concentration in the workplace and it should be noted that the maximum limit established for the Portuguese Highway Code should not be generalized to all professions or tasks [65]. Nevertheless, several Portuguese organizations are governed by the values defined in the Highway Code. For instance, the Collective Bargaining Agreement for the Construction and Public Works Industry that has been in force since April 1, 2010, in clause no. 78 (on the prevention and control of alcoholaemia) considers that it is under the influence of alcohol the worker who, under examination by BrAC, reveals 0.5 g/L or more. For workers under the Highway Code, the BAC provided in that Code is applicable.

### 6.2. Interpretation of Other Psychoactive Substances Results

Regarding the analysis of other psychoactive substances, toxicological analysis can be performed in various biological samples, being more commonly analyzed in oral fluid [83,84,85] and urine [81,83] or in hair [86] and nails [87], given that, although analytically possible, the toxicological results are not relevant to allow for a quantitative interpretation [65]. Indeed, in most cases, only by using blood, serum, or plasma is it possible to truly document the impairment. Therefore, the most correct procedure will be to consider as positive the test that reveals the presence of illicit psychoactive substances above the defined concentrations cut-offs for screening and confirmation tests [88], regardless of the quantitative interpretation the test may provide [89]. Although urine is an important sample for toxicological screening, it is only useful if freshly voided and collection is witnessed and supervised to avoid adulteration. Indeed, the collection facilities should be arranged to prevent adulteration of the specimen as much as possible and adding coloring agents to toilet water has been recommended to reduce the risk [81]. Some authors suggest that all urine specimens taken for drug testing from both the workplace and court settings need to be tested for validity [90]. Nevertheless, urinating is a personal act and most people feel inhibited about being observed in such circumstances by close family members, medical staff, or even sexual partners, and the situation can never be less than humiliating. Furthermore, individuals may not want to disclose pregnancy or venereal diseases. A wide variety of collection devices are available in the market to collect oral fluid for toxicological analysis. A minimum validity test should be performed for oral fluid, such as visual inspection of the sample(s), measurement of oral fluid volume and testing on matrix authenticity through measurement of endogenous biomarkers such as salivary amylase and cortisol [83,84,85].

Hair samples have been gaining popularity. This is by far the most expensive method, but it is claimed to be more secure in the event of legal challenges, to provide conclusive evidence of rebuttal, and is an excellent indicator of addiction, as opposed to occasional use, which may be particularly suitable for pre-employment testing programs. Analysis of nail clippings may be a useful back-up for hair analysis when hair is unavailable [87,91,92]. Useful applications of nail analysis may be where contamination is highly unlikely (i.e., if the fingers do not become contaminated) as is the case during the consumption of tablets or capsules as medication or where the question of time or quantity of use rather than the fact of use is being investigated [92].

The most frequently analyzed substances are amphetamines and derivatives, cannabinoids, cocaine, opioids, and benzodiazepines. Regarding cocaine, immunoassays detect the metabolite benzoylecgonine quite accurately without much concern for false positive or negatives. Nevertheless, the metabolite is inactive and may be present up to 3 days after use, whereas most clinical effects of cocaine occur within 6 to 12 hours of use [26,27]. As for amphetamines, there are many false positives, including antihistamines, decongestants, antidepressants, or acid-blockers, and these may be detected 1 to 3 days after use. Indeed, methamphetamine only differs from pseudoephedrine in a single atom of oxygen. In fact, many methods flowed by clandestine laboratories use *l*-ephedrine (present in stimulant supplements and used for weight loss) or *d*-pseudoephedrine (present in several decongestants drugs) as starting reagents to produce *d*-methamphetamine [93].

Regarding cannabinoids, common over-the-counter analgesics such as ibuprofen and naproxen, as well as the increasing interest in the use of medical *Cannabis*, can increase the probability of cross-reaction with this assay, creating false positives. Many opioids can be missed during routine screening, generating significant false-negatives, and it has been reported that screening is also prone to many false-positive results in the presence of poppy seeds and quinolone antibiotics [13,94]. Indeed, poppy seed paste used in foods could lead to positive urine tests for opioids, even if increased cut-off levels are used and different biomarkers are considered for the differential diagnosis, such as the presence of thebaine to ensure justice for each individual [13,95]. Moreover, if an individual is currently taking a lawful medication containing codeine, the test can suggest that he/she has used heroin.

Nevertheless, it should be mentioned that there are several unscreened substances in existence, such as ketamine, chloral hydrate, gamma-hydroxybutyrate (GHB), psilocybin, mescaline, and cathinones. Moreover, qualitative or semi-quantitative immunoassays may cross-react with other substances, many of which are licit, namely, pharmaceutical drugs [89]. In this case, tests showing the presence of prescription drugs should not be considered positive.

On the basis of the technical limitation of the immunoassay analysis, whenever positive results (i.e., above a predefined cut-off level) emerge at the screening stage, confirmatory tests on the sample must be carried out by a referenced toxicology laboratory, normally by gas and liquid chromatography coupled with mass spectrometry. If the screen results are all negative, no further analysis is necessary. Results of confirmatory analysis are usually not readily available, and even if positive results are obtained, interpretation is required by skilled toxicological professionals in conjunction with a qualified occupation medicine physician [96].

Additionally, of note is that there are many other clinical situations with signs and symptoms similar to psychoactive substance poisoning, and thus definitive diagnosis is only possible through clinical examination. Therefore, a differential diagnosis is needed to find out whether the reason for the positive result can be explained by prescribed medication; some other acceptable reason; or whether it is, in fact, a question of drug abuse [97]. If an occupational physician is not present, the worker should be removed from activities that put his/her or others’ safety at risk and should be properly referred to a health unit. It is underlined that in this case all labor rights, including remuneration, must be ensured for as long as the worker is away from work.

### 6.3. Worker’s Refusal to Undergo Toxicological Analysis

Besides the health professional (or other) who is competent to carry out the sample collection for toxicological analysis, it should be made possible for the worker to request the assistance of a witness, having a defined time to do so, but the test cannot be stopped if the presentation of the witness was not feasible. Refusal to submit to screening tests for psychoactive substance use, although possible, does not imply that the worker is unsuitable for work. When unjustified, the worker may incur any disciplinary offence and, in some enterprises, the refusal leads to the assumption that the worker has a BAC of 0.5 g/L or higher and therefore the employee is unfit/inapt for work. Moreover, it should be highlighted that if a worker is declared unfit by the occupational medicine as a result of psychoactive substance use, it is not a just cause for dismissal or to affect career progression, as clinical results are confidential and as such cannot be made known to the hierarchical superiors or other entities outside the clinical sphere. Only the behavior that may result from the influenced state can be framed according to Article 351 (just cause for dismissal) of the Portuguese Labor Code and as such be subject to disciplinary sanction (and possibly dismissal) due to the unacceptable or breach of established rules.

As a precautionary measure, if a BAC of 0.5 g/L or greater is assumed or presumed, or if positive illicit psychoactive substances results are obtained, the worker will be immediately prevented from working during the remaining daily work period, with the consequent loss of remuneration for such period.

If the worker is found to abuse psychoactive substances in the workplace, it is possible and permissible to initiate disciplinary proceedings against the worker, especially in organizations with internal rules prohibiting the use of psychoactive substances in the workplace.

## 7. Conclusions and Future Perspectives

The use of psychoactive substances in the workplace is a public health problem that influences the safety and health of workers and extends beyond the workplace itself. It is a contentious issue that has moved up the human resources agenda in recent years, and different organizations take diverse approaches to psychoactive substance workplace testing, some of them following a zero-tolerance approach, whereas others have had to develop a more nuanced policy. Such cases may also have insurance and legal repercussions [98,99]. Indeed, workplace psychoactive substance testing is one of the latest components to be added to the discipline of forensic toxicology, which now comprises the triad fields of post-mortem, human performance, and workplace drug testing toxicology [88]. There are several studies that demonstrate that the psychic and motor disturbance due to the consumption of psychoactive substances is in the origin of several work accidents and related economic impacts [12,100,101]. Moreover, testing for psychoactive substances in the workplace, at random or by surprise, has a statistically significant preventive effect in overall professions [102]. Nevertheless, testing in the workplace is a complex topic because it is not often directly regulated by supranational or national law. Only a few countries report legislation that clearly and specifically address the issue of drug testing in the workplace [51].

The participation of workers and their representatives in the design of an occupational health promotion plan and in the definition of policies to be followed undoubtedly plays a decisive role in the implementation of prevention programs against the use of psychoactive substances in the workplace. Human resources policies aimed at promoting worker safety, health, and well-being that integrate worker assistance programs, information campaigns, and other interventions in this field reflect the level of organizational cultures that incorporate concepts and principles of corporate citizenship, encouraging entrepreneurs and managers to good practices, the production of deontological ethics, and codes that value the image of the company or organization and its end products. Workers should also be aware that the employer is committed to creating a work environment that promotes safety and health and that policies and related measures should be applied to all elements of the organizational system so that the individual and collective rights of society will be fully accomplished. Indeed, if on the one hand the use of illicit drugs in the workplace raises issues pertaining to prevention and safety and the responsibility of the various members of staff, on the other hand it also brings into question the interface between work and private life [103]. 

As a prerequisite to testing, the company must have an antidrug program aiming to ensure health and safety, including a written drug testing internal regulation. In this regulation, problems related to the use of psychoactive substances should be considered as health problems and consequently should be treated like other health problems in the workplace regarding temporary disability, sick pay, and other social benefits, especially during periods of rehabilitation treatment.

It is important to clarify that under the law, the screening, counterproof, or confirmatory analysis should not be charged to the worker because they are preventive health and safety activities of companies and the costs must always be supported by the employer. All these measures are stimulating and represent facilitating factors for recovery, although under treatment, the employer must also ensure that the workstation is maintained or transferred to other duties that do not pose a risk to the safety of himself or others without loss of rights or other benefits. Nevertheless, an employee’s failure to successfully complete treatment requirements can eventually result in the termination of their employment.

Nowadays, we are witnessing a paradigm shift from an evolution centered on the treatment and rehabilitation of proven psychoactive substance dependents to a cross-cutting approach through the implementation of a specific regulation that includes (i) prevention of consumption through information actions on health consequences of the use of psychoactive substances; (ii) early detection; (iii) treatment at the enterprise (if it has the means), or through referral to other specialist physician, or to local or regional specialist services (e.g., integrated response centers or alcoholology units) to recover workers in compliance with personal freedom, and these treatments cannot be imposed by coercion; and (iv) socio-professional rehabilitation of the worker, aiming at relapse prevention.

For the full implementation of these objectives, the availability of skilled human resources specifically in occupational medicine with toxicological background and skilled toxicologists in clinical, forensic, and analytical aspects of psychoactive substances, to correctly interpret toxicological results, are needed. Given this reality, fulfilling the purposes of the law, namely, Article 19 of the Portuguese Labor Code, is an almost impossible task. There should be a balance between prevention and repression.

Finally, policies and educational interventions to reduce psychoactive substance consumption in the workplace are needed [104]. These should address awareness to workplace hazards, physician education to promote best practices for treatment, and overdose prevention. In addition, the phenomenon of the new psychoactive substances are also motifs of concern in the workplace that should not be disregarded [51]. Indeed, the consumption of new psychoactive substances poses a significant risk to public health and a challenge to national and international drug policies for occupational medicine, and only very recently have laboratories begun to offer screening and confirmation analysis for new psychoactive substances at lower costs [105].
